# The Novel Data Collection and Analytics Tools for Remote Patient Monitoring in Heart Failure (Nov-RPM-HF) Trial: Protocol for a Single-Center Prospective Trial

**DOI:** 10.2196/32873

**Published:** 2022-06-30

**Authors:** Ankit Bhatia, Gregory Ewald, Thomas Maddox

**Affiliations:** 1 Division of Cardiology Washington University School of Medicine Saint Louis, MO United States; 2 Healthcare Innovation Lab BJC Healthcare Washington University School of Medicine Saint Louis, MO United States; 3 The Christ Hospital Health Network Cincinnati, OH United States

**Keywords:** heart failure, remote patient monitoring, clinical innovation, digital health, mHealth, mobile health, cardiology

## Abstract

**Background:**

Heart failure remains a leading cause of mortality and a major driver of health care utilization. Despite numerous medical advances in heart failure, associated hospitalizations continue to increase, owing largely to suboptimal outpatient management. Remote patient monitoring (RPM) aims to further address this current need in heart failure care by providing data to clinical teams to act pre-emptively to address clinical decompensation. However, to date, RPM approaches using noninvasive home-based patient sensors have failed to demonstrate clinical efficacy.

**Objective:**

The Novel Data Collection and Analytics Tools for Remote Patient Monitoring in Heart Failure (Nov-RPM-HF) Trial aims to address current noninvasive RPM limitations. Nov-RPM-HF will evaluate a clinician co-designed RPM platform using emerging data collection and presentation tools for heart failure management. These tools include a ballistocardiograph to monitor nocturnal patient biometrics, clinical alerts for abnormal biometrics, and longitudinal data presentation for clinician review.

**Methods:**

Nov-RPM-HF is a 100-patient single-center prospective trial, evaluating patients over 6 months. The outcomes will include patient adherence to data collection, patient/clinician-perceived utility of the RPM platform, medication changes including the titration of guideline-directed medical therapy to target doses, heart failure symptoms/performance status, and unplanned heart failure hospitalizations or emergency department visits.

**Results:**

This prospective trial began enrollment in March 2020 and anticipates enrollment completion by June 2022, with trial completion by December 2022.

**Conclusions:**

This trial protocol aims to provide a systematic framework for the evaluation of heart failure RPM strategies, which are currently heavily used but seldom robustly studied. The trial results will help to inform the role of noninvasive RPM as a viable clinical management strategy in heart failure care.

**International Registered Report Identifier (IRRID):**

DERR1-10.2196/32873

## Introduction

Heart failure (HF) remains a leading driver of morbidity and mortality in the United States, affecting 6.5 million Americans [1]. Despite numerous medical innovations in HF over the past decade, per capita rates of HF hospitalizations have actually increased from 2010 to 2017 [2]. This population is expected to increase by 50% by 2030, placing an increasing burden on the US health care system and highlighting the need for innovations in HF care [3]. HF care also carries significant economic implications, with over US $30 billion spent in direct HF care in 2020, primarily driven by HF hospitalizations, and estimated to double by 2030 [4-6].

Effective HF care requires vigilance for indications of clinical HF decompensation. Failure to detect and manage these indicators can result in unplanned emergency department (ED) visits and hospitalizations. Although patients and care teams attempt to detect signs of impending decompensation with conventional techniques such as daily weight measurements, these approaches have not demonstrated consistent clinical efficacy [7].

Optimal HF care also relies on maximizing guideline-directed medical therapies (GDMTs). Several pharmacotherapies such as renin-angiotensin-aldosterone system inhibitors and beta-blockers have demonstrated mortality benefit in patients with HF with reduced ejection fraction (HFrEF) [8,9]. However, consistent provision of these therapies, at maximally tolerated dosing, does not occur [10]. Logistical challenges of conventional outpatient care contribute to this implementation gap. These include reliance on ambulatory visits for medication titration, which often occur months apart, and insufficient vital sign information to allow for medication up-titration between visits.

Remote patient monitoring (RPM) aims to address these gaps in current HF care. RPM is a strategy that allows care teams to monitor and manage patients outside of traditional in-person health care encounters. The strategy could enhance current efforts for more rapid medication titration and avoidance of clinical decompensation and unplanned acute care visits. RPM has demonstrated a reduction in HF hospitalizations, but only by using an invasive hemodynamic patient sensor (CardioMEMS) [11]. RPM strategies using noninvasive patient sensors such as blood pressure (BP) cuffs, scales, and wearable biosensors have reduced cost, lower risk, and greater applicability to the HF population. However, the clinical evidence supporting noninvasive RPM is mixed, with the majority of clinical trials failing to demonstrate a similar clinical benefit [12]. This lack of clinical efficacy in trials is likely attributable to multiple factors, including poor patient adherence [13], lack of clinically actionable patient data and alerts [14], and lack of clinician engagement [15].

The field of noninvasive RPM continues to evolve, with recent advances in data collection and monitoring now demonstrating improved predictive power for clinical decompensation [16]. However, there is a paucity of studies evaluating these emerging tools as part of RPM interventions.

The Novel Diagnostic Tools for Remote Patient Monitoring in Heart Failure (Nov-RPM-HF) Trial aims to address these limitations through the design and evaluation of a novel RPM platform for HF management that incorporates these emerging data collection tools. The trial will evaluate the perceived utility of the RPM platform, its impact on timely GDMT optimization and medication changes, and its impact on unplanned ED visits and hospitalizations.

## Methods

### Overview

The Nov-RPM-HF trial is a single-center prospective study of an RPM platform and its adoption, clinical utility, and ability to optimize GDMTs and reduce unplanned acute care events among patients with HFrEF. Specifically, the trial will evaluate patient adherence with passive and active data collection, patient-perceived usability and utility of the RPM platform to monitor their health status, clinician-perceived usability and utility of the RPM platform for HF management, clinical utility of the RPM clinical alerts, impact of RPM on medication changes and titration of HF GDMT, and impact of HF RPM on clinical outcomes (ED visits, hospitalizations).

Full inclusion and exclusion criteria are summarized in Textbox 1. Inclusion criteria include history of HFrEF with a HF hospitalization or ED visit in the last 12 months. This criteria selects for a HFrEF population with a baseline event rate that mirrors the trial population in the CHAMPION (CardioMEMS Heart Sensor Allows Monitoring of Pressure to Improve Outcomes in NYHA Class III Heart Failure Patients) trial. Such an event rate should allow for increased power to detect clinical outcomes among our study population. Prior noninvasive RPM trials have included a relatively stable population of patients with HF with low clinical event, and this was thought to have contributed to the inability to demonstrate clinical efficacy [13]. End-stage HF with a life expectancy <1 year or requiring advanced therapies including left ventricular assist devices or cardiac transplantation were excluded. Patients with barriers precluding them from obtaining monitored data (eg, wearable defibrillator use preventing ballistocardiograph sensing) were also excluded.

**Inclusion and exclusion criteria.**

**Inclusion criteria**

- Age≥18 years
- HF (heart failure) hospitalization or emergency department visit in last 12 months
- HF with reduced ejection fraction: most recent left ventricular ejection fraction (LVEF) of <50% and at least 1 recorded LVEF of <40%
- New York Heart Association Functional Class II-IV
- Sleeps in same bed ≥4 days per week
- Ambulatory
- Willingness to complete the required surveys, measurements, and study activities

**Exclusion criteria**

- Advanced HF therapies: inotrope therapy, left ventricular assist device or cardiac transplantation
- Wearable defibrillator or other worn device that may affect ballistocardiogram measurements
- End-stage renal disease on chronic dialysis
- Malignancy undergoing active therapy
- Weight >385 lbs at time of enrollment
- Living in a skilled nursing facility or other chronic care facility
- Planned major surgeries or procedures requiring hospitalization in next 6 months
- Hospice care or life expectancy <1 year

Patients were prospectively screened and recruited from the Advanced Heart Failure clinic at the Washington University School of Medicine’s Division of Cardiology. This clinic specifically treats patients with HF, with a focus on implementation of GDMTs and evaluation for advanced therapies including mechanical circulatory assist devices and cardiac transplantation when indicated. The trial was approved by the institutional review board at Washington University for either written or phone-based informed consent. The trial began recruitment in March 2020.

### Ethical Considerations

The trial was approved by the ethics review board of the Washington University Office of Ethics and Integrity, with all information for patient informed consent reviewed. The trial was deemed to be in equipoise with current standard of care for heart failure management.

### RPM Platform

**Figure 1:**
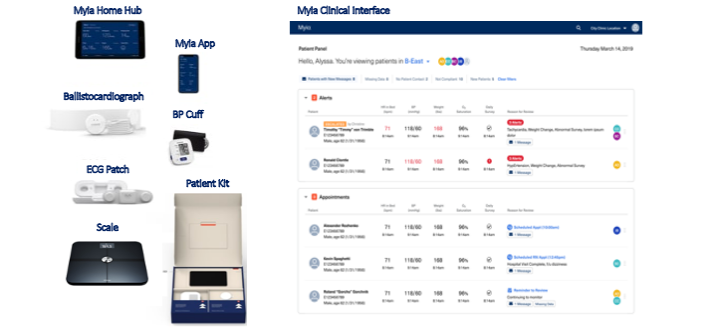
Remote patient monitoring platform used in the Novel Data Collection and Analytics Tools for Remote Patient Monitoring in Heart Failure Trial. BP: blood pressure; ECG: electrocardiogram.

The RPM platform for the trial was provided by a third-party vendor, Myia Health (San Francisco, CA). The platform consists of patient biometric sensors, a digital “Home Hub” tablet to aggregate data from the sensors and transmit to the vendor for analysis, and a web-based clinical interface for clinicians to review data. The clinical interface was co-designed with the advanced HF clinicians to incorporate clinician priorities in its design. To ensure that the RPM platform was optimally designed for clinician and patient needs, Myia conducted clinician and patient interviews (n=51), and incorporated their input into the design of the RPM platform (Figure 1). These interviews identified the following priorities for the prospective RPM platform: home sensors that collected data with minimal or no actions from the patient, optimizing ease-of -use clinical monitoring platform for clinicians with integration into current workflows, and data curation through smart triaging to minimize information overload for the clinical teams.

The RPM platform (Figure 1):

- Patient biometric sensors: Sensors include a weight scale, BP cuff, and a ballistocardiograph. The ballistocardiograph is a pressure-based sensor that is placed under a patient’s bed mattress. It measures ballistic forces related to heart and lung movement to derive heart rate (HR), respiratory rate (RR), and other associated physiologic parameters. Importantly, the ballistocardiograph is always “on” and only requires the patient to lie in bed to collect data, thus minimizing the data collection burden for patients.
- Home Hub tablet: An Android (Google) LTE-enabled tablet loaded with the Myia application serves as a data aggregation and transmission point for collected vital sign sensor data. It also includes a user interface to solicit, collect, and transmit patient-reported outcomes.
- Clinical interface: The clinician interface displays sensor data for all patients currently enrolled in the trial. The interface includes a data visualization workspace that juxtaposes longitudinal health data (vital signs, symptoms) with event data (ED visits, hospitalizations) to aid in clinical management. Patient data is prioritized for clinical review using alerts. Alerts are customized based on patient-specific thresholds (eg, vital signs outside of predefined range), with deviations outside normal range triggering an alert. Additionally, among patients on submaximal GDMTs, medication-specific alerts are included to allow for rapid HF medication up-titration.

### Study Protocol

Following enrollment, study participants have the Home Hub and sensors shipped to their homes. Once the kit is delivered, the technical support team will reach out to assist with home monitoring and sensor setup.

As part of the study, patients will take measurements daily from all study sensors. The first 7 days for each patient will be a run-in period where patient data is collected. If sufficient data is collected and transmitted, study participants will transition to the active management phase. Sufficient data is defined as transmission from both BP cuff/weight scale and ballistocardiograph sensors in at least 4 of the 7 days. If minimum requirements are not met, patients are continued in the run-in period for an additional week (up to 3 weeks maximum). Technical support will contact patients to determine if lack of transmission represents a technical issue versus nonadherence. If patients were unable to meet minimum data requirements after 3 weeks, then they were disenrolled from the study.

Following run-in period completion, study participants will enter the 6-month active management phase of the study. A designated research nurse is responsible for daily monitoring of the RPM clinical interface. This includes monitoring the RPM clinical interface daily for clinical alerts, contacting patients to assess clinical status in response to alerts, and escalating to the patient’s HF cardiologist if clinical status warrants management changes. This research nurse will also be responsible for contacting study participants for issues with data transmission or poor adherence to patient data collection.

**Figure 2:**
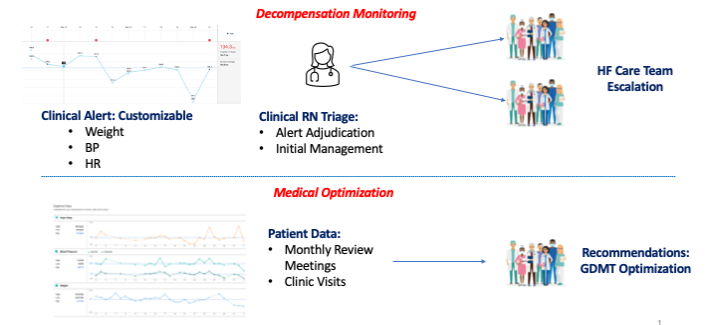
Remote patient monitoring platform monitoring strategies. BP: blood pressure; GDMT: guideline-directed medical therapy; HF: heart failure; HR: heart rate.

On a monthly basis, enrolled patients will have their clinical data reviewed by a multidisciplinary team including a triaging nurse and an HF cardiologist. The purpose of these reviews is to assess participant’s clinical status and adherence to data transmissions, and to identify opportunities for optimized medical management, including maximizing GDMT. RPM platform monitoring is outlined in Figure 2.

#### Clinical Alerts and Thresholds

Several clinical monitoring alerts will be used prospectively during the management phase of the study. Each alert has default thresholds that are customizable to individual study participants. Absolute threshold alerts will be turned on by default in all patients, while GDMT alerts can be opted-in by the clinical team for patients deemed to be on submaximal GDMT. Default values are shown in Textbox 2. All alerts are required to be addressed within 24 hours by the monitoring nurse and escalated to the clinical team if indicated. Actions taken by the clinical team in response to the alerts were documented.

**Remote patient monitoring clinical interface parameters with default values.**

**Absolute thresholds alerts**

High and low thresholds for weight, blood pressure (BP), and heart rate (HR). Thresholds include both default absolute values and patient-specific deviation from baseline values.

- Weight
  >3 lbs over 1 day
  >5 lbs over 3 days
- BP
  Systolic blood pressure (SBP)>200 mmHg
  SBP>180 mmHg each day for 3 consecutive days
  SBP<80 mmHg
- HR
  HR<35 bpm average in 1 night
  HR>150 bpm

**Guideline-directed medical therapy (GDMT) alerts**

These alerts are generated for patients on submaximal GDMT. They are designed to permit rapid up-titration of GDMT, customized based on drug class.

- Beta-blocker
  HR>80 bpm (nightly average) and SBP>100 mmHg for 3 consecutive days
- Angiotensin-converting enzyme inhibitor, angiotensin receptor blocker, angiotensin receptor neprolysin inhibtor, and mineralcorticoid receptor antagonist
  SBP>110 for 3 consecutive days

#### Outcomes

Trial outcomes were designed to both directly evaluate the perceived utility of the RPM platform and to assess its impact on clinical outcomes.

##### Patient Adherence to Data Collection and Transmission

Patient data collection adherence will be measured for all home BP cuff, scale, and ballistocardiograph sensors as percentage of days with data transmission from each device over the 6-month trial duration. “Minimally-useful data profile” is defined as the percentage of weeks with at least 4 days of data transmission from both an *active* (BP cuff or weight scale) and *passive* (ballistocardiograph) sensor.

##### Patient-Perceived Usability and Utility of RPM Platform

Patients’ assessment of the RPM platform utility and usability will be assessed through a series of surveys (survey timeline in Multimedia Appendix 1). Surveys will be conducted at 2, 4, and 6 months. Surveys will have scaled numerical responses 1 to 7, with 17 total questions. Questions will assess RPM platform components (scale, BP cuff, ballistocardiograph) usability, platform setup, and the platform’s perceived utility in augmenting clinical care. The sum of the numeric responses for each question will combine into an “overall utility score.” This score will be assessed for change throughout the study duration for each participant. Surveys will be reviewed at the time of submission by the research nursing group. Patients will be contacted regarding any significant RPM platform concerns. Scores will also be analyzed to assess for RPM platform strengths/weaknesses and to evaluate for change in perceived utility over the trial duration.

##### Clinician-Perceived Usability and Utility of RPM Platform

Along with patients, clinicians will also be assessing the RPM platform’s utility and usability through a series of surveys (survey timeline in Multimedia Appendix 1). Surveys will be conducted at 3 and 6 months, with scaled numerical 1 to 7 ratings of 14 components of the RPM platform, with a focus on the clinical interface.

##### Clinical Utility of RPM-Generated Clinical Alerts

The number of absolute threshold clinical alerts (Textbox 2) generated by the RPM platform will be reported, along with its accompanying clinical action. Clinical actions include medication changes (adding/holding medications), scheduling a follow-up appointment, advising immediate medical care (ED visit or hospitalization), or no action. The clinical utility of each absolute threshold alert will be measured as the percentage of clinical alerts prompting clinical action and categorized by physiologic measure (ie, weight, BP, and HR).

##### Medication Changes

Clinician-directed changes will be tracked during trial duration for the following HF medication classes: beta-blockers, angio-converting enzyme (ACE)/angiotensin receptor blocker (ARB)/angiotensin receptor-neprilysin inhibitors (ARNIs), magnetic resonance angiographies (MRAs), hydralazine, nitrates, and loop diuretics. The distance to target dose will also be assessed for GDMT medications, beta-blocker, ACE/ARB/ARNI, and MRA drug classes. Based on previously established methodology [10], baseline doses of each of these drug classes will be the classified percentage of target dose: 0% to 25%, 25% to 50%, 50% to 75%, and 75% to 100%. This target dose category will be compared for each participant between 0 and 6 months to assess for changes in GDMT dosing. Change in proximity to target dose over trial duration will be compared to that of matched controls (see “Statistical Considerations” for matching methodology). For all drug classes, the absolute number of medication changes will be recorded and compared to that of the matched controls.

##### HF Symptoms and Performance Status

Study participants’ symptoms and performance status will be evaluated using the Kansas City Cardiomyopathy Questionnaire (KCCQ-12) at 0, 3, and 6 months (Multimedia Appendix 1), with absolute results and change over study duration reported.

##### HF Clinical Events

The study’s primary clinical end point is HF hospitalizations. Secondary clinical events include other unplanned hospitalizations and ED visits. Clinical events will be obtained through monthly electronic health record review by the research team and with monthly patient surveys. All hospitalizations will be adjudicated by the clinical team as either HF or other unplanned hospitalizations. Clinical end points will be compared to those of matched controls.

#### Statistical Analysis

Patient medication changes and clinical events will be compared to 100 matched historical controls. Controls will be patients with HFrEF matched on an HF hospitalization in the past year, age within 5 years, sex, race, and comorbidities (coronary artery disease, diabetes mellitus, hypertension, renal disease).

Statistical analysis for clinical end points will be in comparison with matched controls. For survey data, including RPM platform utility and patient symptom assessments, these will be evaluated for change over the trial’s 6-month duration. Continuous variables will be assessed via sample *t* tests, and categorical variables will be assessed via chi-square tests.

#### Power Calculation

##### Medication Changes

This trial is powered at 99% to assess for 50% more medication changes in the study arm compared to historical controls. We will use a type I error of 0.05, assuming Poisson distribution [17]. This medication change rate was selected based on prior work in the CHAMPION trial, in which monitored patients had over double the number of medication changes (n=2468 for 270 patients vs n=1061 for 280 patients; *P*<.001) [18].

##### HF Symptoms and Performance Status

This trial is powered at 84% to assess for a 6-point increase (SD 20) in KCCQ scores for trial participants over the trial duration. We will use a type I error of 0.05. This improvement in KCCQ scores was based on the Minnesota Living with Heart Failure symptom assessments improvements observed in the CHAMPION trial [19].

##### HF Hospitalizations

Regarding HF hospitalizations, Nov-HF-RPM is underpowered for this end point. Power calculation for clinical HF events was conducted using the baseline event rates observed in the CHAMPION trial given similar inclusion criteria [19]. Effect size was estimated to be roughly 50% that observed with CardioMEMS. Using type 1 error of 0.05, the study would require 1054 recruited patients (with 1054 matched controls) to obtain 80% power. We will nonetheless plan to collect this clinical end point to better understand if there is a positive trend with RPM. Future studies with larger patient populations will be required to obtain adequate statistical power for hospitalizations.

## Results

This prospective trial began enrollment in March 2020 and anticipates enrollment completion by June 2022, with trial completion by December 2022.

## Discussion

Optimal HF care requires the ability to effectively monitor and treat impending clinical decompensation so as to prevent ED visits and hospitalizations. RPM aims to address the current gaps in outpatient clinical care. To date, the only RPM strategy using an invasive hemodynamic sensor (CardioMEMS) has demonstrated the ability to reduce HF hospitalizations [11]. RPM strategies using noninvasive biometric sensors are a potential lower risk and lower cost approach to RPM. However, prior trials evaluating noninvasive approaches to RPM have not demonstrated clinical efficacy [12,20].

Several limitations of noninvasive RPM strategies are thought to contribute to the lack of demonstrated efficacy in these prior studies. These include poor patient adherence to monitoring, a lack of clinically actionable patient data and alerts, and a lack of clinical buy-in. The design of the Nov-RPM-HF study seeks to overcome these limitations.

Poor patient data collection adherence was observed in the majority of noninvasive RPM clinical trials and is thought to have contributed to neutral outcomes [12]. Both patient-perceived utility and ease-of-use of RPM platforms are believed to be contributing factors to nonadherence. Accordingly, Nov-HF-RPM will assess if improved adherence can be observed through the use of *passive* sensors for patient data collection that do not require direct patient action. The trial will also directly compare data collection adherence between *passive* (ballistocardiograph) and *active* sensors (BP cuff, scale). The ballistocardiograph adds a novel component to the home sensor collection, with the potential to augment adherence and provide continuous nightly data for HR and RR. To date, several studies using ballistocardiograph data-derived algorithms have demonstrated the ability to effectively classify compensated versus decompensated disease states in HF [21,22]. However, the role of the ballistocardiograph in an HF management strategy has not been studied.

A lack of clinically actionable RPM alerts has been commonly observed in prior noninvasive RPM studies. A systematic review of noninvasive RPM trial clinical alerts revealed patients were contacted only 39% (range 29%-52%) for follow-up and management changes [14]. Furthermore, RPM alerts with high false-positive rates have previously led to unnecessary office visits and hospitalizations [23]. Nov-RPM-HF will assess clinical utility of all absolute threshold alerts based on if there is a corresponding clinical action. Alert utility will be broken down by alert type (weight, BP, HR) to assess comparative utility of each physiologic measure.

Although seldom measured in RPM clinical trials, adoption and buy-in from the clinical team is essential for maximal and effective RPM use. Factors affecting clinical adoption include perceived utility of RPM-provided patient data and integration into existing clinical workflows [15]. Nov-RPM-HF evaluates a clinician-designed RPM platform, and clinical utility will be assessed through structured surveys, broken down by RPM platform components, and evaluation of clinical actions motivated by RPM alerts.

Nov-RPM-HF will provide an important contribution to the field of RPM for patients with HFrEF. Its design attempts to overcome prior limitations of prior RPM strategies and determine its impact on patient engagement and satisfaction, clinical satisfaction, and HF medical optimization. Insights from the study will inform the future role of noninvasive RPM as a viable HF clinical management strategy.

## Appendix

Multimedia Appendix 1 Study survey timeline for patients and clinicians.

## Abbreviations

ACE: angio-converting enzyme
ARB: angiotensin receptor blocker
ARNI: angiotensin receptor-neprilysin inhibitor
BP: blood pressure
CHAMPION: CadioMEMS Heart Sensor Allows Monitoring of Pressure to Improve Outcomes In NYHA Class III Heart Failure Patients
ED: emergency department
GDMT: guideline-directed medical therapy
HF: heart failure
HFrEF: heart failure with reduced ejection fraction
HR: heart rate
KCCQ-12: Kansas City Cardiomyopathy Questionnaire
MRA: magnetic resonance angiography
Nov-RPM-HF: Novel Data Collection and Analytics Tools for Remote Patient Monitoring in Heart Failure
RPM: remote patient monitoring
RR: respiratory rate
