# Supplementary material for: The Novel Data Collection and Analytics Tools for Remote Patient Monitoring in Heart Failure (Nov-RPM-HF) Trial: Protocol for a Single-Center Prospective Trial
Source: JMIR Res Protoc. 2022 Jun 30;11(6):e32873. doi: 10.2196/32873 (PMC9284360; doi:10.2196/32873)
Supplement: Multimedia Appendix 1 [file resprot_v11i6e32873_app1.docx]

|  |  | **Screening/Baseline Visit** | **Run-In Period** | **After Run-In Period** | | | | | | | | | | | | | | | | | | | | | | | | |
| --- | --- | --- | --- | --- | --- | --- | --- | --- | --- | --- | --- | --- | --- | --- | --- | --- | --- | --- | --- | --- | --- | --- | --- | --- | --- | --- | --- | --- |
|  |  |  |  | **1 Day** | **1 Week** | **2 Weeks** | **3 Weeks** | **1 Month** | **5 Weeks** | **6 Weeks** | **7 Weeks** | **2 Months** | **9 Weeks** | **10 Weeks** | **11 Weeks** | **3 Months** | **13 Weeks** | **14 Weeks** | **15 Weeks** | **4 Months** | **17 Weeks** | **18 Weeks** | **19 Weeks** | **5 Months** | **21 Weeks** | **22 Weeks** | **23 Weeks** | **6 Months** |
| **SURVEYS** | | | | | | | | | | | | | | | | | | | | | | | | | | | | |
| Patient | Eligibility Survey | 🗸 |  |  |  |  |  |  |  |  |  |  |  |  |  |  |  |  |  |  |  |  |  |  |  |  |  |  |
| Patient | Medication Change/ Compliance Survey |  |  | 🗸 |  |  |  | 🗸 |  |  |  | 🗸 |  |  |  | 🗸 |  |  |  | 🗸 |  |  |  | 🗸 |  |  |  | 🗸 |
| Patient | KCCQ-12 |  |  | 🗸 |  |  |  |  |  |  |  |  |  |  |  | 🗸 |  |  |  |  |  |  |  |  |  |  |  | 🗸 |
| Patient | Hospitalizations Survey |  |  |  |  |  |  | 🗸 |  |  |  | 🗸 |  |  |  | 🗸 |  |  |  | 🗸 |  |  |  | 🗸 |  |  |  | 🗸 |
| Patient | Patient Performance Survey |  |  | 🗸 | 🗸 | 🗸 | 🗸 | 🗸 | 🗸 | 🗸 | 🗸 | 🗸 | 🗸 | 🗸 | 🗸 | 🗸 | 🗸 | 🗸 | 🗸 | 🗸 | 🗸 | 🗸 | 🗸 | 🗸 | 🗸 | 🗸 | 🗸 | 🗸 |
| Patient | Check-in 1 Survey |  |  |  |  |  |  |  |  |  |  | 🗸 |  |  |  |  |  |  |  |  |  |  |  |  |  |  |  |  |
| Patient | Check-in 2 Survey |  |  |  |  |  |  |  |  |  |  |  |  |  |  |  |  |  |  | 🗸 |  |  |  |  |  |  |  |  |
| Patient | Check-in 3 Survey |  |  |  |  |  |  |  |  |  |  |  |  |  |  |  |  |  |  |  |  |  |  |  |  |  |  | 🗸 |
| Patient | Post Visit Review Survey |  |  |  |  |  |  |  |  |  |  |  |  |  |  | 🗸 | | | | | | | | | | | | |
| Clinician | Patient Data Review Survey |  |  |  |  |  |  | 🗸 |  |  |  | 🗸 |  |  |  | 🗸 |  |  |  | 🗸 |  |  |  | 🗸 |  |  |  | 🗸 |
| Clinician | Mid-Point/End-Point Survey |  |  |  |  |  |  |  |  |  |  |  |  |  |  | 🗸 |  |  |  |  |  |  |  |  |  |  |  | 🗸 |
| Clinician | Physician Visit Review Survey |  |  |  |  |  |  |  |  |  |  |  |  |  |  | 🗸 | | | | | | | | | | | | |

**Multimedia Appendix 1:** Study Survey Timeline for Patients and Clinicians
